# Using crime script analysis to understand wildlife poaching in Vietnam

**DOI:** 10.1007/s13280-020-01498-3

**Published:** 2021-03-18

**Authors:** Julie Viollaz, Barney Long, Cao Tiến Trung, Josh Kempinski, Benjamin M. Rawson, Hoàng Xuân Quang, Nguyễn Ngọc Hiền, Nguyễn Thị Bích Liên, Cao Tiến Dũng, Hoàng Thương Huyền, Renée McWhirter, Nguyễn Thị Thùy Dung, Meredith L. Gore

**Affiliations:** 1 United Nations Office on Drugs and Crime, Vienna, Austria; 2 Global Wildlife Conservation, Austin, TX 78767 USA; 3 Vinh University, 182 Le Duan Street, Vinh, Vietnam; 4 Fauna & Flora International, Vietnam Progarmme, 118 Tu Hoa, Tay Ho, Hanoi, Vietnam; 5 WWF-Vietnam, No. 06, Lane 18, Nguyen Co Thach Rd., Nam TuLiem Dist., Hanoi, Vietnam; 6 Center for Environment and Rural Development, Vinh University, Vinh, Vietnam; 7 The Crawford Research Institute, 278 Crawford Street, Toronto, ON M6J 2V8 Canada; 8 Department of Geographical Sciences, University Or Maryland, 2181 LeFrack Hall, College Park, MD 20740 USA

**Keywords:** Conservation criminology, Environmental criminology, Protected areas, Situational crime prevention, Snares, Wildlife crime

## Abstract

Poaching can contribute to the failure of biodiversity conservation efforts and inflict diverse harms on human livelihoods. We applied crime script analysis to the case of snare poaching—an illegal hunting activity—in three Vietnamese protected areas. Our goal was to enhance the understanding about the opportunity structure underlying snare poaching to advance the suite of community-based crime prevention activities. We analyzed crime scripts for three types of poachers across nine stages of the poaching process using expert-based elicitation with 13 workshop participants in Vinh, Vietnam, 2018. Five stages were similar, clustered toward the early stages, and two were different, clustered around middle crime stages. Analysis produced systematic crime-specific insight about the procedural aspects and requirements for poaching from preparation to hunt to selling one’s catch. Stages identify multiple entry points to apply prevention techniques and match techniques with different types of snare poaching or poachers. Although this research focused on protected areas, the interdisciplinary approach applied herein may be adapted to other conservation contexts.

## Introduction

The loss of biodiversity from poaching, a form of illegal hunting, can have long-term effects on forests’ ability to support human and animal populations that rely on these ecosystems (Corlett 2007), resulting in, for example, empty forest effects (Antunes et al. 2016). Illegal hunting may occur in a wide range of circumstances and for a large number of reasons; it may be driven by, for example, economic motivations, culture and tradition, incomplete awareness of rules and laws, restrictions on traditional access to resources, crop guarding, lack of engagement during rule-setting, and/or large-scale criminal enterprises (Kahler and Gore 2012).

Conservation biologists and conservation social scientists often study illegal hunting within the context of *why* individuals comply or do not comply with formal or informal conservation rules (e.g., Peterson et al. 2019; Shirley and Gore 2019) as well as traditional subsistence pathways, for which the criminalization of the behavior may not necessarily have been constructed in consultation with local peoples. Extant research explores what species are at risk from illegal hunting and the actors and networks involved (e.g., Phelps et al. 2016), the role that hunting plays as a normalized and sustainable livelihoods issue for rural people highly dependent on natural resources (e.g., Kahler and Gore 2012), the legitimacy of poaching behavior being criminalized, stigmatization from criminalizing poaching, and partial controllability of hunting-related laws and norms (see Gore 2017). Nongovernmental organization reports complement the peer-reviewed literature, estimating the scale of the “snaring crisis,” quantifying its impacts on diverse wildlife and indigenous human populations, describing regional differences in responses to snaring, and suggesting recommendations for policymakers and other practitioners (e.g., Belecky and Gray 2020). And yet, unsustainable hunting continues with profound implications for ecosystem function and human livelihoods (Belecky and Gray 2020). Gaps remain in our understanding about how to effectively and efficiently apply criminogenic solutions to the conservation problem of illegal hunting practices (e.g., Dobson et al. 2019). One implication of this gap is that unintended consequences of criminogenic interventions aimed at preventing illegal hunting may go undetected (Dobson et al. 2019) and the problem will persist with irreversible consequences.

Illegal snare hunting is ubiquitous within the forests of Southeast Asia (Gray et al. 2017a, b; Belecky and Gray 2020). The activity continues unabated due to a number of factors, such as the ease of acquiring construction materials, snares’ effectiveness at capturing wildlife, and ability to be deployed across different ecosystem types. Individuals engaging in illegal snaring generally have a low chance of being caught and punished and perceive that illegal snare hunting is not a serious activity (Watson et al. 2013). Local people may be aware of the illegality of activities, but prohibitions may lack legitimacy and there may be a disconnect between authorities and society regarding legal definitions and their interpretation. Trapping with snares is known to be motivated by financial gain and non-pecuniary benefits such as social esteem and enjoyment (versus poverty per se) (Macmillan and Nguyen 2014); commercial illegal wildlife trade fuels the activity (Belecky and Gray 2020).

Snare poaching remains a persistent conservation issue with myriad connections to the global “wildlife crime” crisis (UNODC 2016; Gray et al. 2017a, b). Although efforts have been made by different agencies, organizations and sectors to reduce illegal wildlife harvest from snaring, these have been largely unsuccessful (Belecky and Gray 2020; MacMillan and Nguyen 2014). New(er) conceptual approaches for thinking about the topic are emerging, such as green, environmental, or conservation criminology (e.g., Gore 2017; Kurland et al. 2017; South and Wyatt 2011), human ecology (e.g., Dobson et al. 2019), and socio-environmental systems thinking (e.g., Carter et al. 2017). These approaches broaden thinking about interventions for conservation based on a more holistic understanding of human behavior (UNODC 2016). Beyond considering *why* harms occur, we can also explore *how* they occur and better adapt crime prevention tools developed for street crimes such as automobile theft to the context of conservation harm such as illegal snare hunting.

Currently, in Southeast Asia, basic insight is known about how the *operating environment* shapes behavioral opportunities for illegal hunters to hunt and where and when they do so. Operating environments may be dynamic, localized, and influence conservation interventions’ ability to achieve outcomes (e.g., Gore and Knuth 2009); they also create the environmental conditions that enable harms or crime to occur. This is a different focus than answers to the important questions of *why* people engage in snaring, *why* they are motivated and driven to snare, or *why* law enforcement authorities are not as effective as they might be. Rather, different understanding about the sphere of activity within which illegal snare hunting occurs can help inform localized efforts to reduce poaching in some of the world’s most critically endangered ecoregions important for rare, newly discovered, and endemic species such as the saola (*Pseudoryx nghetinhensis*) (Hardcastle et al. 2004). This assistance is derived from criminogenically derived data about methods, techniques, and decision points over the course of the illegal snare hunting process; it is not intended to be a replacement for conservation-based insight of the problem, but rather a force multiplier to existing solutions. For example, Belecky and Gray (2020) provided an overview of the snaring crisis and provided recommendations for patrolling and snare removal as well as legal regulation of snaring, emphasizing that snares contribute to a wildlife extinction crisis while also impacting ecosystems that support human well-being. Criminological insight can serve as a force multiplier to insights in the gray literature, such as Belecky and Gray (2020).

## Characterizing an operating environment that enables illegal snaring

Here, we apply environmental criminology theories and methods to help clarify “situational factors” of the operating environment (situational determinants and choice-structuring properties in criminology) that enable illegal snaring of wildlife in Vietnam’s Annamite Mountains, an area experiencing high snaring pressure. Poaching using snares made from common items such as bike wires or winches is inexpensive, easy to use, lethal, and carries almost no risk of detection. Poaching is a major direct threat to Vietnam’s wildlife and snaring has contributed to the failure of many conservation efforts, such as the extinction of the Javan rhinoceros (*Rhinoceros sondaicus*) from Vietnam in 2010 (Brook et al. 2014). Snaring is implicated in the rapid decline of many other species in Vietnam, and the country is at the center of the regional snaring crisis; snare use also increases human exposure to species carrying zoonotic diseases (Belecky and Gray 2020). Law enforcement authorities can be hesitant to arrest poachers from minoritized or indigenous groups, for whom wildlife was historically a major protein source and more recently a means to fulfill livelihood needs (Brunner 2012; Tanalgo 2017). However, these authorities, particularly mobile ranger teams, are involved in protecting poaching sites and preparing violence reports. Oftentimes, it is challenging for researchers to access such violence reports because information in the reports has personal information, is part of an ongoing investigation, or is otherwise deemed private/sensitive and not shared. Our interdisciplinary approach—applying crime science theory to a conservation issue—attempts to provide *complementary* insight about the sequence of decisions and acts necessary to commit a specific crime or harm at a specific time, rather than build knowledge about why people commit crime or harm generally (Cornish and Clarke 1986). This information can supplement violence reports and other frameworks for assessing the impacts of wild meat hunting practices (e.g., Dobson et al. 2019; Belecky and Gray 2020). By understanding this “thought and action” sequence and the situational factors that drive it, one can more precisely target techniques to prevent, constrain, or disrupt the activity. This can be accomplished through crime script analysis (see Dehghanniri and Borrion 2019).

## Environmental criminology, crime triangles, and crime scripts as tools for conservation

We intentionally adopt and use terminology from the field of criminology since these techniques were originally developed and used for classic crime prevention. Environmental criminology analyses crime patterns through the lenses of space, place, and time, while recognizing the role(s) that environmental and situational factors (e.g., belonging to the surrounding physical space) play in crime commission (Brantingham and Brantingham 1995; Wortley and Mazerolle 2008). One of its foundational theories is the routine activities approach (Cohen and Felson 1979), which states that crime occurs because of the confluence of a suitable target (e.g., valued animal) and an offender (e.g., poacher) in the absence of capable guardians against crime (e.g., ranger).

**Fig. 1:**
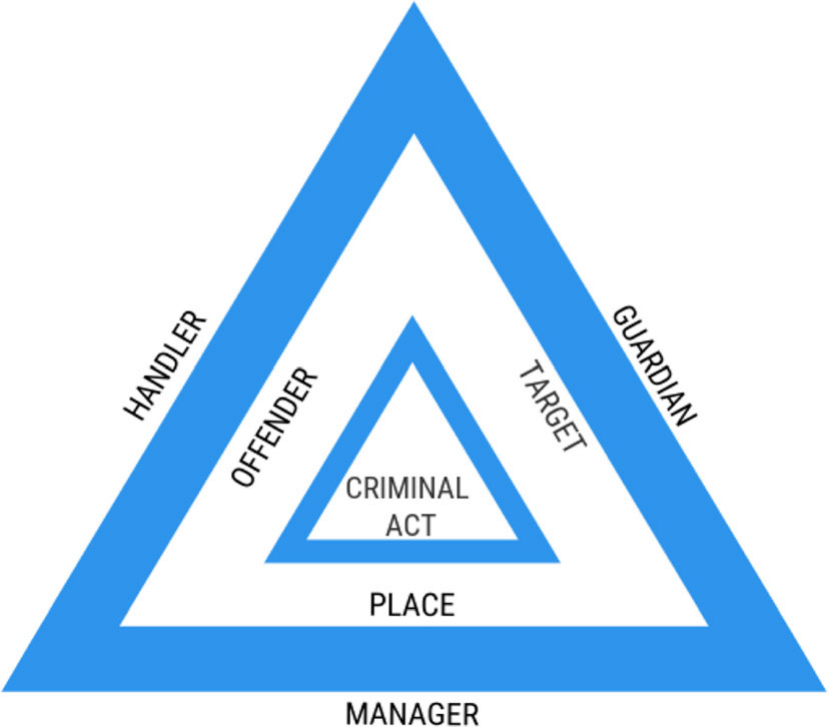
The crime triangle (adapted from Eck 2003)

“Crime triangles” model the necessary elements for a crime to occur and the actors (e.g., guardians) whose presence acts as a crime deterrent (Eck 2003a, b) (Fig. 1). Offenders can sometimes be “controlled” by other people known as handlers. Targets and victims can sometimes be protected by other people, known as guardians, while managers are those who “control” places (Cohen and Felson 1979; Eck 2003a, b). Exploring the relationships between offenders, targets, and places can direct the location, time, or condition of strategies for guardians to discourage the commission of crime. A crime script (Cornish and Clarke 1986) is one tool for clarifying the interrelationship between variables in the crime commission process and intervention points that guardians can focus on to deter potential offenders. Illegal snare hunting is a crime in Vietnam’s protected areas, according to the rule of law (i.e., Decree 32/2006/ND-CP). Neither in this paper nor during the research process were we advocating for criminalization of illegal snare hunters in the Annamite Mountains; rather, we apply a theoretical framework to the real-world problem of illegal snare hunting to enhance scientific understanding of the suite of community-based approaches to this wildlife conservation issue. There are heterogeneous environmental justice, indigenous rights and historical dimensions to wildlife and protected area laws in Vietnam that connect to matters of morality and blame, responsibility and injustice, crime and punishment, unregulated power, and governmental indifference; Belecky and Gray (2020) offered a summary of some of these issues from a conservation perspective. Brisman (2007) offered a review of crime-environment relationships and environmental justice from a green criminology perspective. This work expands on topics discussed by these authors.

A crime script analysis offers one step-by-step review of how a specific crime is committed, identifying the complete sequence of decisions and actions prior to, during, and after the crime and the links between them (Cornish 1994). The tool helps contextualize the relationship between harm and the environment in ways that distinguish fear of crime *on* the environment versus *in* the environment, which is important for conservation (e.g., Belecky and Gray 2020). The analysis considers nine stages or steps, which we describe here using the context of poaching and the vernacular of criminology:

1. *Preparation* Acquiring necessary tools, selection of co-offenders, as well as agreeing on the selected locations to engage in offending.
2. *Entry* Gaining access into selected locations where the poaching is to be undertaken.
3. *Precondition* Enabling the commission of a crime, such as waiting at the location for place managers to leave.
4. *Instrumental precondition* Identifying suitable targets.
5. *Instrumental initiation* Closing in and approaching the target, or animal to be poached.
6. *Instrumental actualization* Engaging with the target, such as isolating the animal for poaching.
7. *Doing* Carrying out the intended crime such as poaching the animal.
8. *Post-condition* Leaving the crime setting.
9. *Exit* The decisions that need to be made post-crime commission such as selling of poached animal.

## Strengths and limitations of crime scripts for conservation

A crime script analysis is not typically viewed as a complete account of crime commission, but can be used as evidence to craft crime prevention interventions for that specific crime across the stages (Cornish 1994; Viollaz et al. 2018), including those that may be implemented early (Rowe et al. 2013). Ideally, several intervention points, interventions, and actors to intervene are identified and used in concert, especially in cases like illegal snare hunting where deterring prospective offenders is even harder given the high rewards and the ease of laying snares and catching wildlife (Ayling 2013). In terms of disaggregating types of actors to intervene, crime scripts can help identify non-official interveners (known as informal guardians in criminology) who might otherwise be overlooked. Informal guardians may be community or family members with ties to potential offenders and are often individuals who are not directly involved in crime prevention or enforcement but deter crime by their mere presence in a crime-prone location or by providing information on offenders to direct interventions effectively (Reynald 2011). Because they are informal, they can leverage social relationships in ways that formal guardians cannot, and should not for ethical reasons, and can therefore influence the social contexts or remote causes of crime (Ayling 2013). These informal guardians become especially key in places like Vietnam where formal guardianship mechanisms are often ineffective (MacMillan and Nguyen 2014).

**Fig. 2:**
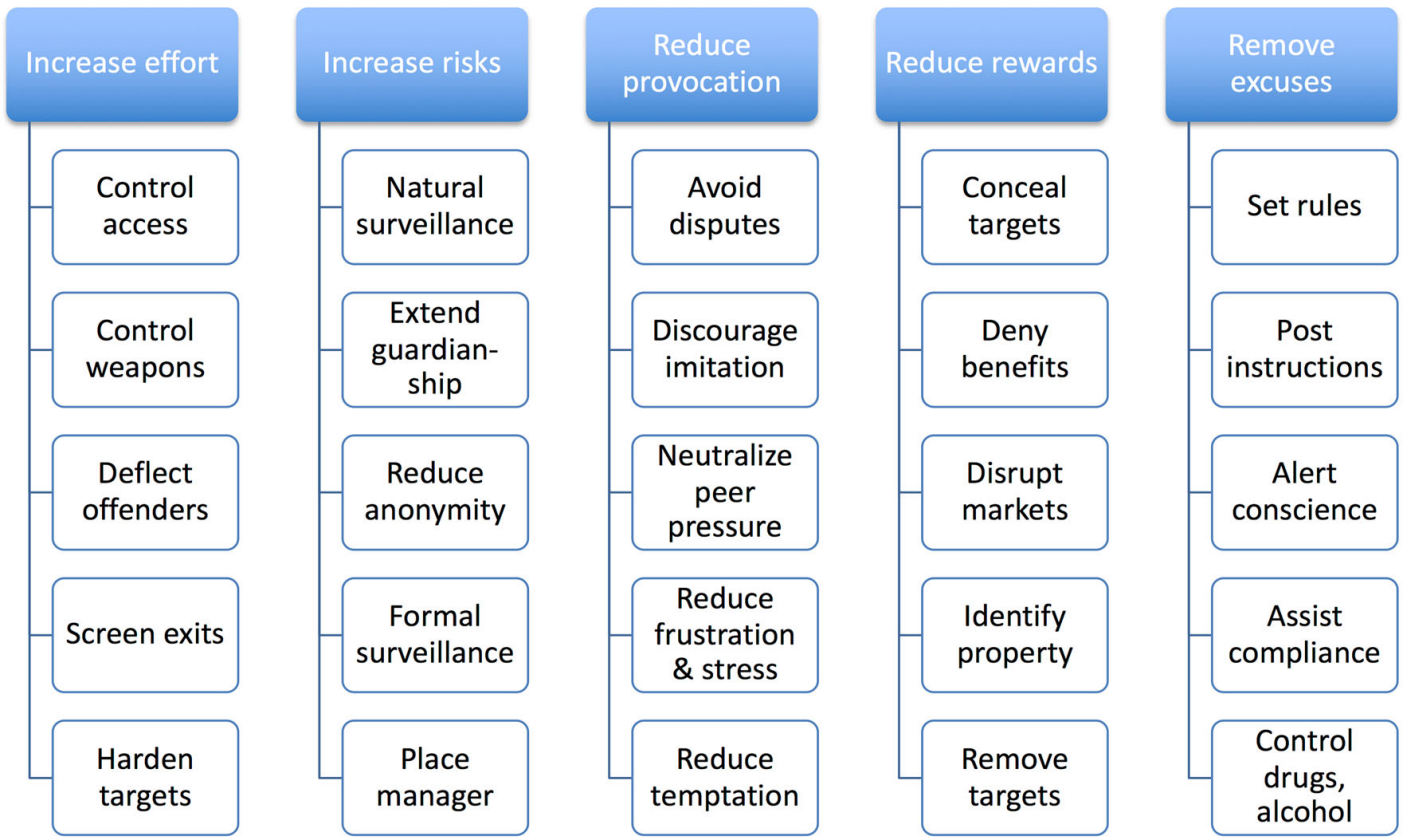
The 25 techniques of situational crime prevention (adapted from Clarke 2008a)

Delineating the opportunity structure underlying illegal snare hunting using crime scripts also makes it possible to leverage targeted opportunity reduction tools such as Situational Crime Prevention (SCP) techniques (Fig. 2; Clarke 2008a, b, see Viollaz 2016 for an application to conservation problems), 5I’s framework (Ekblom 2011), or crime prevention through environmental design (Crowe 2000). SCP techniques, for example, specifically alter the situational factors enabling opportunities for crime. SCP involves increasing the risk or effort required to commit crime (for example, making it harder for hunters to acquire the snare materials to hunt), reducing the rewards from crime, and deterring an offender from making a decision to commit a specific crime at a particular time and place, rather than changing their willingness to engage in crime generally (Clarke 1980) (which is what operations designed to produce livelihood alternatives do) (Ferraro and Kiss 2002). Situation-specific interventions that locally deter criminal behavior are important for conservationists because long-term projects to change mindsets to address wildlife crime generally may only realize their full potential well after a target species has gone extinct or be implemented at coarser scales that do not reach all offenders (e.g., Brooke 2014). Ultimately, crime scripts contribute to an immediate localized solution that can be applied to stop poaching at source, while other interventions both along the supply chain (e.g., market control, technical support for procuracy and courts) and over time (e.g., demand reduction campaigns) are implemented in tandem to other facilitating elements of wildlife related crime for lasting impact.

Since wildlife crime is driven by a complex array of factors (Travers et al. 2019), crime scripting is ideally viewed as a complementary, and not replacement, tool for reducing risks to people and wildlife from illegal snare hunting. It does not pretend to offer a long-term or comprehensive review of all connections between crime and the environment (Brisman 2007). While some scholars have questioned the utility of crime scripts in terms of completeness and whether they are solely addressing problems of the past (Cornish 1994), they have been applied successfully to wildlife crime. For example, Viollaz et al. (2018) used script analysis to identify the financial crimes associated with rhino horn trafficking. The Poaching Diaries (Lemieux 2020) included example of crime scripting to understand diverse wildlife crimes like redwood burl poaching and amber mining. We acknowledge that these tools and the solutions/conclusions drawn from them are only as good as the quality and authenticity of the data used. Using these tools in a sustainable, ethical, and socially accountable manner requires carefully assessing data quality and triangulating all data for maximum accuracy. When used in combination with other data (e.g., Belecky and Gray 2020), the aperture on analytically relevant variables can be focused for more effective conservation.

As of yet, crime scripts have not been applied to the context of illegal snare hunting in Vietnam. Considering nine steps of a crime commission process challenges the overall assumption in conservation that poaching is a single event (e.g., mapping a poaching event in a database such as the African Wildlife Poisoning Database). The purpose of this research was to enhance understanding of the criminogenic dimensions of illegal snare hunting, not to stigmatize sustainable use by traditional hunters. To our knowledge, there has been no empirical study in the conservation literature of the process of illegal snare hunting from this criminological perspective. This interdisciplinary approach helps close the gap on how this conservation crime problem can be understood and thus how solutions might be innovated. In the discussion, we synthesize crime script insights with crime prevention techniques specifically intended to engage both informal and formal guardians as responders to poachers, illustrating the contemporary best practice of “plural policing” which recognizes the functions of enforcement and crime prevention may be delivered not only by institutions of, but also by other actors through, above, beyond, and below, government (Ayling 2013).

## Materials and methods

### Study sites, ecosystem, and local people

We focused on illegal snare hunting in three protected areas in Vietnam’s Annamites ecoregion: Pù Mát National Park, Quảng Nam Saola Nature Reserve, and Thừa Thiên-Huế Saola Nature Reserve—areas already designated as high priorities for conservation by diverse conservation organizations and authorities (e.g., Belecky and Gray 2020). Although no law clearly prohibits carrying materials into protected areas, the minimum penalty for any type of hunting by snares in a protected area is between $11 000 and $13 000 (Belekay and Gray 2020).

These three sites are part of the Greater Annamites ecoregion (Olson and Dinerstein 1998) which is dominated by the Annamite Mountain Range along the border of Vietnam and Laos. The Vietnamese, or eastern flanks of the Annamites, are characterized by very wet evergreen broadleaf forest (Baltzer et al. 2001). The fauna of the ecoregion are distinct and characterized by endemic species such as the saola (*Pseudoryx ngethinhensis*), large-antlered muntjac (*Muntiacus vuquangensis*), Annamite striped rabbit (*Nesolagus timminsi*), and doucs (*Pygathrix *sp.). The rarity of the regions’ taxa is largely brought about by the intensive threats facing the ecoregion. Research has consistently illustrated the main threat to the large mammal community is ubiquitous and non-selective snaring (Gray et al. 2017a, b; Belecky and Gray 2020)

PùMát National Park is situated in Nghệ An province within the Northern Annamites landscape. The park borders Laos to the west and a development frontier of villages and agriculture to the east. The park is 91 200 ha with a buffer zone of 86 000 ha, dominated by villages of Thai and Kinh indigenous peoples. A small number of Dan Lai households live within the core zone of the park; this ethnic minority has a long history of fleeing military pursuers. The park rises from 100 m to 1 841 m a.s.l., although 90% of the park is below 1 000 m (SFNC 2001). The western boundary of the park is comprised of the Annamite ridge with steep sided valleys of four major rivers extending to the east. Sixty two percent of the core zone of Pù Mát is primary forest with a further 30% being disturbed forest (SFNC 2001).

The Thừa Thiên-Huế and Quảng Nam Saola Nature Reserves span the Bach Mã—Hải Vân Mountains running east–west along the border between Thừa Thiên-Huế and Quảng Nam provinces. This is the wettest area of Vietnam receiving up to 8 000 mm of precipitation annually and forms the southern distribution of many Annamite endemic species including saola, red-shanked douc (*Pygathrix nemaeus*), and Edward’s pheasant (*Lophura edwardsi*) (Long et al. 2005). The area is predominantly primary very wet evergreen broadleaf forest with steep sided valleys running north and south on either side of the east–west mountain ridge. The Thừa Thiên-Huế Saola Nature Reserve is 15 342 hectares in size and bordered to the north by villages of Tà Ôi and Katu people; Katuic people traditionally held religion centered around spirits of the forest and have been subjected to policies of mobilization and sedentarization (Arhem 2009). The Quảng Nam Saola Nature Reserve is 15 486 hectares in size and bordered to the south by villages of the Katu people.

### Data collection

Qualitative data to build the crime triangle and crime script were derived using a sequential data collection strategy (Singleton et al. 1993). Data were collected during a two-week workshop on environmental criminology and its application to wildlife crime that was held at Vinh University, Vietnam, in March 2018 (i.e., iterative expert-based elicitation; Trochim and Donnelly 2001). Participants [n = 13, female = 9, year born range = 1974–1996, ethnic groups = Kinh (92%), Mông (8%)] were a mix of local academics/practitioners working on conservation issues in the study sites and students doing fieldwork for biological sciences degrees; in this regard they are conservation “experts.” Both groups had worked or were currently working with local hunters and communities on the ground in the study sites for various conservation research projects, including long-term projects on hunting and alternative livelihoods where they interacted directly with hunters. They had lived in these communities during fieldwork and therefore had first-hand experience of local hunting practices, making them well suited, apart from the hunters themselves, to describe how snaring took place. They were recruited through Vinh University’s Center for Environmental and Rural Development based on having the qualifications and attributes described above and being able to participate in all research activities.

All participants were workshopped by the lead author through a series of environmental criminology lectures and practice activities to build common understanding over the course of two full work weeks from 9 am to 5 pm. Environmental criminology lectures focused on the crime theories and concepts (e.g., opportunities theories, deterrence, crime concentration, situational determinants, and choice-structuring properties), crime analysis methods (e.g., problem-oriented policing, the crime triangle, crime scripts), and crime prevention tools (e.g., guardianship, risk perception, situational crime prevention). Each lecture was followed by a practical exercise to apply the concepts learned to the Vietnam context for snare hunting. These are the data on which the crime script is based. The workshop participants all sat together in the same room for lectures and used whiteboards and handouts on the conservation criminology concepts learned to work on the practical exercises as a group. The final results of the exercises were reviewed and revised where needed by the group the following day.

The expert group was asked to use their knowledge of the local context (e.g., livelihood needs and vulnerabilities, historical use of protected area lands) and lived experience about how and where hunters set snares to identify the offenders, targets, and location of illegal snare hunting in the study sites as well as their *modus operandi* and to identify opportunities for community-led crime prevention interventions. The focus was on hunters’ choices and actions since hunters are the ones directly catching wildlife and therefore their behavior is requisite for preventing snaring. The demand for wild meat at the consumer end is, of course, a larger driver of the trade, and demand reduction programs on that end of the trade chain are necessary to fully tackle the snaring problem so hunters are further disincentivized by a lack of customers. Our focus, though, was on immediate measures to curtail hunter’s ability to hunt, regardless of demand, versus their underlying motivations to snare.

Participants were encouraged to consider the characteristics of handlers, guardians, and managers who could be valuable actors for such initiatives. The research lead guided participants through discourse that first enabled collective delineation of a crime triangle and then qualitative and iterative analysis of the stages of crime commission (i.e., (1) preparation, (2) entry, (3) precondition, (4) instrumental precondition, (5) instrumental initiation, (6) instrumental actualization, (7) doing, (8) post-condition, and (9) exit). She collated feedback, summarized findings back to the participant group, and facilitated collective revision on the nine stages when needed in order to produce a critical evaluation of the crime script. Where opinions differed on the steps taken to illegally hunt with snares, the majority opinion with the most evidence to back it up was retained. The process of iterative discourse to build consensus about the stages of poaching commission over multiple days helped support the validity and reliability of the conclusions for this particular conservation context (Trochim and Donnelly 2001). This form of didactic expert elicitation is commonly applied in conservation when there are gaps in knowledge and a common conceptual design is necessary to facilitate interdisciplinary thinking (Morss et al. 2018; Tobi and Kampen 2018). The materials, methods and analysis used in this research were approved as exempt by The Human Subjects Protection Program at Michigan State University’s Institutional Review Board approved the project under 45 CFR 46.101(b)2 (Study ID STUDY00000372).

## Results

Workshop participants first identified three types of hunters with the potential to illegally hunt and differentiated between their processes for illegal hunting in PAs. Although we acknowledge concepts such as crime, offender, guardian and deterrence are social constructions that are essential for effective conservation (see Massé et al. 2018), we do not condone blanket use of such terminology. Hunters were described as either subsistence (thợ săn nghiệp dư) or professional hunters, the latter split into two categories: inside (thợ săn chuyên nghiệp địa phương) and outside professional hunters (thợ săn chuyên nghiệp từ nơi khác đến). Subsistence hunters conduct hunting seasonally in their free time and derive their primary income from agriculture. Importantly, subsistence hunters also collect non-timber forest products (NTFPs) from the PAs. These hunters were unlikely to have sophisticated snaring technology (e.g., used simple tools and snares) and since their hunting was secondary to their main occupation as farmers, they spent less time in the forest and tended to be less selective in where they hunted, indiscriminately targeting wildlife. Professional hunters used snare hunting as their primary livelihood strategy. They used sophisticated snares and tools, knew the best places to hunt and were excellent trackers and navigators of the forest. Inside professional hunters lived in close proximity to the PA in which they set snares, while outside professional hunters traveled into the PAs from outside the local vicinity. The proportion of outside relative to inside professional hunters was unclear. Participants’ disaggregation of three hunter types during this stage was integral to future discussions and was a foundational result from this research.

**Fig. 3:**
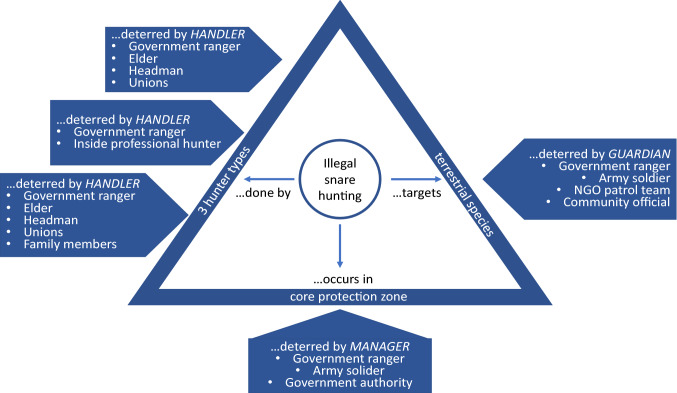
An expert-based crime triangle of illegal snare hunting in three Vietnamese protected areas combined three types of hunters

Participants then delineated crime triangles for each type of hunter, as well as guardians and place managers available to help prevent illegal snare hunting, which were combined into a single crime triangle (Fig. 3). Environmental and conservation criminology recognize the potential for third parties to be active participants in developing and implementing crime prevention interventions (Reynald 2011). In fact, there is usually a diverse array of third parties, or informal guardians (vs. formal guardians such as park rangers) whose potential to prevent conservation crime has been underutilized. Our results indicate this is also true for conservation problems like illegal snare hunting. For example, workshop participants described how inside professional and subsistence hunters acted as gatekeepers for outside professional hunters because they competed for hunting-related resources and insiders had a stake in preventing outsiders from using those resources. Headmen, elders, and local unions also functioned as makers/enforcers of norms at the village level and therefore could act as handlers for inside professional and subsistence hunters. They are able to use their positions of authority and respect in their communities to approach these hunters and ask for their compliance for the good of the community. Some Youth Unions, acting as community liaisons with authorities, were identified as playing a significant role in discouraging local families from hunting by using social pressure. For example, youth group members would volunteer with the families, helping them with planting or other household needs. While doing so, they would openly discuss their beliefs about nature conservation with the families, including the hunters. Hunters often felt a pressure to desist from snaring as a result of their relationships with these youths and the help they were receiving.

All of these actors are guardians of wildlife through their “handling” of offenders and their presence in or near key locations for snare hunting (e.g., place managers). Law enforcement personnel, mainly rangers, were identified by participants as serving as handlers on all “sides of the crime triangle,” but chronic motivational issues, limited patrol effort and coverage, low arrest rates, and poor incentivizing from government authorities suggested low overall crime prevention potential. Based on results from the typology of hunters and crime triangle, workshop participants created three crime script tracks, one each for outside professional hunters, inside professional hunters and subsistence hunters (Table 1).

**Table 1:**
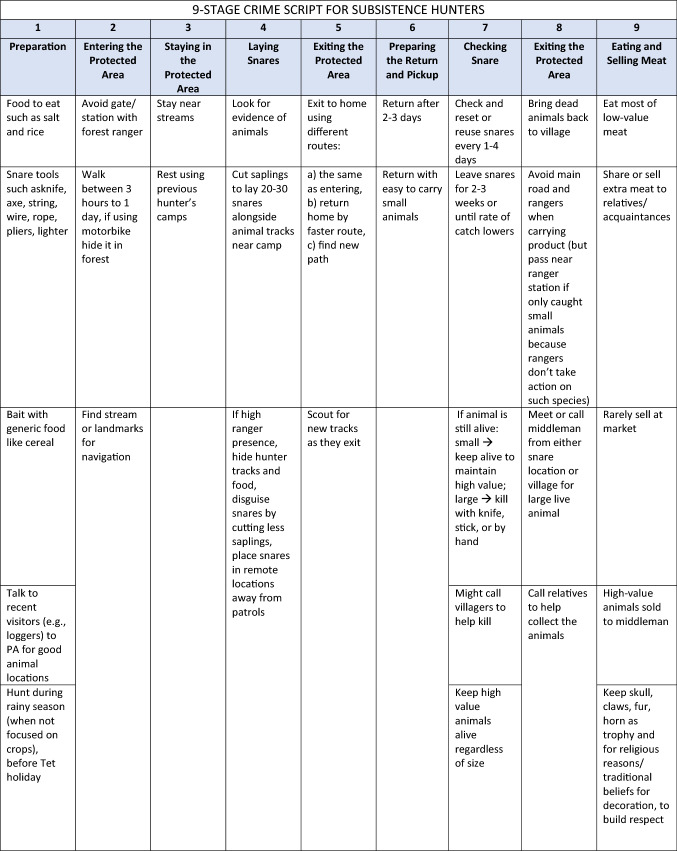

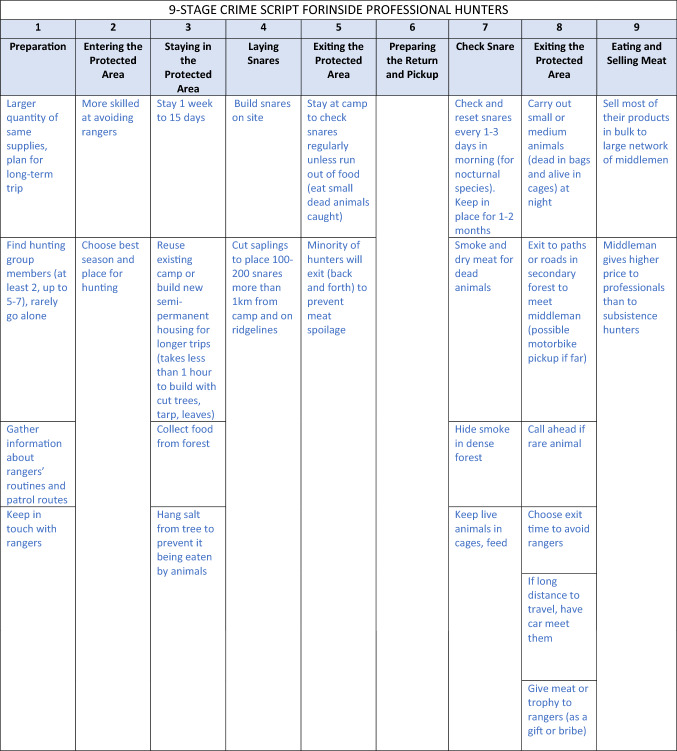

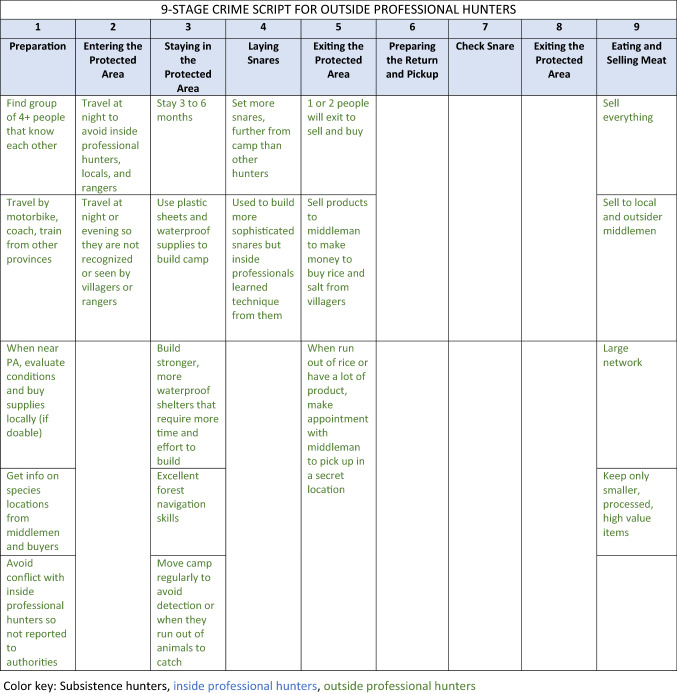
Crime script of illegal snare hunting in protected areas in the Annamite Mountains

A consolidated crime script included each hunter type and compared and contrasted the differences in illegal hunter routines and *modus operandi* by hunter types across each of the nine steps of snare poaching (see Table 1 for the specifics for each hunter type). Participants agreed that each of the nine stages of crime commission were relevant across all hunter types. Five commonalities emerged across hunter typologies, tclustered mostly at the beginning and end of the crime commission process. Stages 4–7 were considered to be the most distinct among hunter types. With regards to commonalities, all illegal hunters:

1. prepared for hunting trips by assembling supplies and gathering intelligence on best places to hunt, although their sources of information varied from rangers to self (Stage 1: preparation);
2. entered the PA and built a camp, although their entry tactics varied by time and place (Stage 2: entry);
3. laid snares; some exited the PA after laying snares and returned at a later date, while others remained and checked snares for several days or weeks (Stage 3: precondition);
4. removed caught wildlife from the PA using methods dependent on catch size and volume (Stage 8: post-condition); and
5. processed, consumed, traded, or sold wildlife depending on catch volume and hunter type (Stage 9: conclusion).

Two “middle script” stages clearly diverged among hunter types: Stage 4 (instrumental precondition, or laying snares) and Stage 5 (instrumental initiation, or exiting the PA). Hunter types differed in terms of the number of snares they would set, whether or not they would exit the PA while they were waiting for snares to catch wildlife, and estimated distance they would set snares relative to their camp site. Understanding these differences is essential to targeting interventions appropriately: these characteristic “patterns of life” help to distinguish the subsistence from the non-subsistence hunters, which is often a goal of law enforcement and criminal justice officials when they focus their operations. These authorities are then able to exercise discretion and often choose to condone subsistence hunting and target enforcement efforts on professionals.

## Discussion

The negative impacts of snaring in protected areas on biodiversity conservation and livelihood preservation are well known in the scientific literature and by on-the-ground conservation practitioners (Robinson and Bennett 2000; Beckley and Gray 2020). These stakeholders are well aware of the tension between overexploitation and sustainable use of biodiversity. Using interdisciplinary approaches like conservation criminology (Gibbs et al. 2010) (that includes expertise from natural resource management, criminology, and risk and decision science) for thinking about the problem of illegal snare hunting opens up new solution spaces that may enhance the sustainability of interventions and spotlight sustainable use, particularly when used alongside technical information about snaring such as Spatial Monitoring and Reporting Tool (SMART) data. Researchers and practitioners may also find methods and approaches discussed here in this case study relevant to other types of conservation crime—activities that violate the rule of law such as illegal fishing, but also activities that damage aquatic resources or disperse species harmful to the environment.

The crime triangle delineated by workshop participants identified multiple stakeholders serving as guardians, both formal and informal. Developing a crime triangle and crime script was especially helpful for identifying places and spaces where informal guardians, such as family members or religious leaders, could best connect with illegal hunters (Bhagwat et al. 2011) and work to prevent the activity. For example, in addition to law enforcement authorities, local hunters were identified as playing a role in preventing illegal snare hunting. This provides an alternative pathway to “full-on” law enforcement responses that can sometimes be perceived as heavy-handed and alienate communities, or work as a first or second line of defense to bolster existing but under-resourced law enforcement efforts. The potential for local guardians, both formal and informal, to have a larger role in preventing illegal snare hunting (i.e., poaching offenders) is broad–both within and outside PAs. Self-policing can also be essential for addressing poaching when the activity is diffused across ecosystems or where limited resources are available for monitoring and enforcement (Peterson et al. 2019). Information about who and where informal guardians live, work, and play can identify entry points for new collaborations or partnerships.

Analysis of crime scripts identified five stages of the crime commission process held mostly in common (e.g., Stages 1, 2, 3, 8, 9) and two divergent (e.g., Stages 4, 5), across three types of hunters with different *modus operandi*. These similarities and differences have implications for on-the-ground interventions particularly those that are focused on types of snares (e.g., removal of single-species target snares, multi-species target snares, or electronic snares) (Beckley and Gray 2020). For example, insight may help enable informal guardians and others conservation stakeholders to consider new strategies to prevent poaching or focus resources on stages of the crime commission process or on specific types of hunters to achieve maximum impact. If the goal is to avoid targeting subsistence hunters and focus on professional hunters, the scripting process helped delineate differences in hunting routines that can help authorities identify and avoid subsistence actors. For example, although all hunter types engage in fairly similar preparation stages, only subsistence hunters are known to be very active before the Tet holiday, when they are not overly focused on their agricultural crops (Table 1, Stage 1).

Since crime prevention and optimizing limited resources are common goals of law enforcement authorities, we chose to consider the results from this crime script through the lens of the SCP framework in order to identify opportunities to increase the risks and increase the effort (Clarke 1980) associated with illegal snare hunting in the study sites and thereby deter offenders. The crime script provides the opportunity to consider SCP interventions that either: (1) capitalize on the crime script stages held in common and pool resources for intervention at common times and places for snaring or, (2) focus on key actors based on the divergent methods used by the different hunter types. We discuss examples of both below, recognizing they occur within a broader context of calls for increased resources to combat illegal snare hunting; changes in laws governing purchase, sale, transport and consumption of wildmeat; and strengthened demand reduction campaigns (Beckley and Gray 2020). Our discussion ideally supports others in their efforts to analyze crime scripts for conservation.

First, Stage 2 involves the point in the crime commission process where hunters enter the PA. Outside professional hunters were associated with the temporal tactic of navigating entrance at night whereas subsistence hunters were associated with the geographic tactic of using streams and topography to navigate during the day. Artificial intelligence-based law enforcement patrolling strategies such as PAWS (Fang et al. 2017), leverage insight about these important differences in “temporal and geographic nodes” to design patrol plans that are most likely to deter hunters and lead to arrests or confiscations, as appropriate.

At this stage in the crime commission process, many opportunities exist to support local formal and informal guardians in efforts to *control access* to PAs. For example, rangers could encourage community members entering the PA to collect NFTPs to act as whistleblowers and report new access points they discover so rangers can monitor them more closely. Generally speaking, informing could be made easier with a dedicated wildlife hotline for anonymous reporting and media time could also be provided to NGOs for educational campaigns.

Second, Stage 9 involves the exit from the crime commission process. Although the snaring has already occurred by this point, opportunities do exist to increase screening at exits through checkpoints, record keeping, and mandatory reporting of suspicious vehicles on primary roadways adjacent to PAs to apprehend middlemen with wildlife in their possession or hunters as they exit the PA with their tools or caught wildlife. With enough certainty, celerity, and severity of punishment at this stage (the three key components for deterrence to work), a deterrent effect would emerge whereby the certainty of getting caught would deter potential offenders from engaging in the crime commission process in the first place.

Third, Stages 2 and 4 in the crime commission process involve periods of time when hunters are preparing to enter a PA or assembling teams to embark on hunting activities. These stages present opportunities to deflect offenders before they set snares, reduce their anonymity and make it harder to engage in snaring without detection, and discourage imitation from other community members who are considering snaring. Many hunters have strong relationships with rangers and so have knowledge of patrol patterns. Although this knowledge is primarily used to evade detection, these preexisting relationships involve trust and legitimacy, which can be leveraged to change certain hunters’ behavior through positive social pressure in informal interactions, before rangers and hunters potentially encounter each other in the PA. Rangers could choose to visit known inside professional hunters’ houses regularly, and check-in to ensure no hunting preparations are ongoing and make them aware that they are under surveillance. This same technique can also be used by headmen and community elders who can leverage their respect among the community to dissuade potential hunters from snaring when they see hunting preparations ongoing in their communities or hear of new potential recruits who want to start snaring.

Crime script analysis systematically identified patterns in the crime commission processes across hunter types (Table 1). In this regard, we found our application of crime scripting to the case of illegal snare hunting in Vietnam to be theoretically and practically appropriate. Convergent spaces (e.g., Stages 1, 2, 3, 8, 9) offer insight for efficient development of interventions to impact the greatest number of hunter types, for example, targeting the season where the greatest number of hunters are in the forest or the common physical locations were crime opportunities exist (e.g., Stage 3: precondition), like where hunters routinely sell their catch. Inside and outside professional hunters may reuse camps set up by previous hunters or from prior hunting excursion while camp destruction is a common activity of rangers. These camps are another example of convergent physical locations for intervention (e.g., Stage 4: instrumental precondition) and can provide natural surveillance opportunities for guardians and contribute to the increased risks strategy of SCP. In such convergent locations, patrolling rangers and informal guardians who enter the PA to collect non-timber forest products, could engage in joint efforts to remove or mark hunter campsites from within PAs. Destroying camping supplies is one additional strategy for interdiction, but it can carry high costs and negative reaction from the public and should be used with caution and intention. Ultimately, local experts are best positioned to determine if incentives, capacity-building, entitlements, or conscription-type mechanisms are most appropriate at preventing illegal snare hunting (Ayling 2013); suggestions here illustrate possibilities that could be implemented in collaboration with other partners so undue burden is not imposed on local stakeholders.

Our insights about the crime commission process for different types of illegal hunters are cross sectional in nature. Importantly, we know that the *modus operandi* of wildlife offenders can shift over time and in response to diverse factors such as local guardians’ patrols (i.e., influencing Stage 6: instrumental actualization). Continued attention to the nine stages of the illegal snare hunting process would help identify trends over time and inform recalibrations of crime prevention or crime response activities. Criminologists often advocate for comparing crime scrips to see where non-enforcement interventions could be used to complement and support standard models based on arrests to deter crime. Or, crime scripts can help map out how partnerships among intervening actors can be used to target multiple stages in the script, leveraging skills, resources and mandates of different organizations and agencies (Lemieux 2020).

The conservation community is innovating in response to the current wildlife crime crisis. However, it is reasonable to assume that wildlife crime offenders will become more experienced. As specific offenses are successfully committed by offenders, offender learning will likely occur in a variety of ways including performance and practice rehearsing, identifying flaws in technique, neutralizations of risks, and overcoming the obstacles and barriers encountered while offending (Cornish 1994). Criminologists know that in order to prevent crime, the “preventers” must understand the crime environment as least as well as the offenders, and preferably better (Cornish 1994). Environmental criminology insights about crime prevention help offer this detailed understanding of the how, where, and when of wildlife crime to innovate in preventing it and adjust as offenders adapt to new prevention measures. This research helps illustrate this potential, using the case of illegal snare hunting in Vietnam’s Annamite Mountains. In identifying snare hunter types, crime triangle characteristics, and distinguishing similarities and differences in hunters’ choices and behavior with crime scripts, we help advance the knowledge base necessary to design effective interventions for a key conservation challenge.
